# Research Letter: Effect of antivirals for COVID-19 on the mortality of older adults dispensed treatment with lithium, antipsychotics, antidepressants, anxiolytics and hypnotics

**DOI:** 10.1177/00048674231217700

**Published:** 2023-12-12

**Authors:** Osvaldo P Almeida, Amy Page, Frank M Sanfilippo, David B Preen, Christopher Etherton-Beer

**Affiliations:** 1 Medical School, WA Centre for Health & Ageing (M577), University of Western Australia, Perth, WA, Australia; 2 School of Allied Health, University of Western Australia, Perth, WA, Australia; 3 School of Population and Global Health, University of Western Australia, Perth, WA, Australia

## Introduction

The COVID-19 pandemic has impacted well-being and mortality worldwide. Older adults and people living with mental health disorders have been disproportionally affected by severe illness and death (Fond et al., 2021), making them a priority population for the implementation of preventive and treatment strategies. Although vaccines have contributed to decrease COVID-19-related deaths, vaccine uptake has been variable and is reportedly low among individuals with mental disorders (Nguyen et al., 2022). Thus, effective treatment of COVID-19 may be particularly helpful to prevent disease-related complications among people who are also receiving treatment for the management of mental health symptoms.

Molnupiravir and nirmatrelvir plus ritonavir treatment reduce COVID-19-related hospitalisation and death, even among non-vaccinated individuals (Hammond et al., 2022; Jayk Bernal et al., 2022). The effect of these agents on mortality in people dispensed medications for the treatment of mental health disorders is not known. An early systematic review concluded that antipsychotics more than doubled the risk of COVID-19-related death (Vai et al., 2021), and preliminary evidence suggests a similar association for anxiolytics, but less likely for antidepressants (Nemani et al., 2021; Oskotsky et al., 2021). At this stage, it is unclear whether exposure to antiviral treatment for COVID-19 modifies such risk.

We aimed to determine the proportion of adults aged ⩾60 years dispensed antiviral treatment for COVID-19 among those who were also dispensed lithium carbonate, antipsychotics, antidepressants, anxiolytics and hypnotics in 2022. We also sought to determine how the dispensing of COVID-19 antivirals affected all-cause mortality among individuals dispensed psychotropic medications.

## Methods

### Study design, setting and participants

We used the 10% Pharmaceutical Benefits Scheme (PBS) sample for the year 2022 provided by Services Australia (Mellish et al., 2015). This database contains information on medicines dispensed by all community pharmacies, and private and PBS public hospitals. The de-identified random sample of 10% of the Australian population aged ⩾60 years included data on age, sex, date of death (if applicable) and medications. The latter were mapped to the corresponding codes of the Anatomical Therapeutic Chemical (ATC) classification system.

The Human Research Ethics Committee of the University of Western Australia reviewed and approved the study (2022/ET000372).

### Study measures

We evaluated the dispensing of antiviral drugs for COVID-19 and death. The antiviral drugs were molnupiravir (Lagevrio – ATC code J05AB18 – www.pbs.gov.au/info/news/2022/03/lagevrio-molnupiravir-pbs-listing) and nirmatrelvir/ritonavir (Paxlovid – ATC code J05AE30 – www.pbs.gov.au/info/news/2022/04/paxlovid-nirmatrelvir-and-ritonavir-pbs-listing), which were approved for the treatment of COVID-19 in early 2022. The 1761 participants who died before 1 March (date of PBS approval for molnupiravir) were excluded, but subsequent deaths were counted (causes of death were not specified).

The dispensing of lithium, antipsychotics, antidepressants, anxiolytics and hypnotics were the exposures. We also retrieved data on age (in years) and sex. The unweighted Rx-Risk Comorbidity Index, a measure of burden and mortality risk based on exposure to medications (Pratt et al., 2018), was calculated following published algorithms, but excluded anxiety, bipolar disorder, depression and psychotic illness (the presence of mental disorders is derived from the dispensing of anxiolytics, lithium, antidepressants and antipsychotics, which are the exposures of interest in this study).

### Statistical analyses

Descriptive statistics were used for discrete data (counts and proportions) and numerical data (mean and standard deviation). The test of proportions and *t*-tests were used to compare crude differences between groups. Multivariate logistic regression was used to examine the association between mortality and psychotropic drugs and COVID-19 antivirals, adjusting for age, sex (male reference) and the simplified Rx-Risk score, including an interaction term between psychotropics and antivirals. The interaction term represents a ratio of a ratio and should not be interpreted as a direct measure of association – the results are reported to highlight the statistical significance of the interaction (*p* < 0.01). Results are summarised by the odds ratio (OR) and respective 99% confidence interval (CI). Alpha was set at 1% and probability tests were two-tailed.

## Results

The study included 549,025 people aged 60 years or over (291,975 females, 53.18%) dispensed medications during the year 2022 (mean age = 72.17 years, SD = 8.60) (Table 1). COVID-19 antiviral treatment was dispensed to 41,362 (7.53%) individuals, lithium carbonate to 1408 (0.26%), antipsychotics to 15,844 (2.89%), antidepressants to 128,773 (23.45%), anxiolytics to 30,808 (5.61%) and hypnotics to 32,979 (6.01%). There were 14,010 (2.90%) deaths between March and December 2022. Antiviral COVID-19 treatment was dispensed more frequently to people dispensed antipsychotics (mean percentage difference = 3.47%, 99% CI = [2.82%, 4.12%]), antidepressants (mean difference = 2.08%, 99% CI = [1.85%, 2.31%]), anxiolytics (mean difference = 1.97%, 99% CI = [1.54%, 2.41%]) and hypnotics (mean difference = 3.25%, 99% CI = [2.80%, 3.70%]) compared with people not dispensed the respective psychotropic medication. There were more deaths among those dispensed antipsychotics (mean difference = 14.16%, 99% CI = [13.41%, 14.92%]), antidepressants (mean difference = 2.10%, 99% CI = [1.95%, 2.25%]), anxiolytics (mean difference = 2.03%, 99% CI = [1.72%, 2.34%]) and hypnotics (mean difference = 2.33%, 99% CI = [2.02%, 2.63%]) compared with people not dispensed these medications.

**Table 1.** Characteristics of the 10% PBS random sample of Australians aged 60 years or over during 2022 according to the dispensing of psychotropic medications.

	Population *N* = 549,025 *n* (%)	Lithium *N* = 1408 *n* (%)	Antipsychotics *N* = 15,844 *n* (%)	Antidepressants *N* = 128,773 *n* (%)	Anxiolytics *N* = 30,808 *n* (%)	Hypnotics *N* = 32,979 *n* (%)
Age in years, mean ± SD	72.17 ± 8.60	69.63 ± 7.65*	74.52 ± 10.08*	72.79 ± 8.92*	72.78 ± 8.93*	74.70 ± 9.03*
Female	291,975 (53.18)	815 (57.88)*	9884 (56.70)*	82,858 (64.34)*	20,002 (64.92)*	21,437 (65.00)*
Rx-risk score, mean ± SD	3.32 ± 2.24	3.53 ± 2.31*	4.18 ± 2.52*	4.09 ± 2.43*	4.24 ± 2.46*	4.29 ± 2.52*
COVID-19 antivirals	41,362 (7.53)	120 (8.52)	1728 (10.91)*	11,755 (9.13)*	2895 (9.40)*	3492 (10.59)*
Died	14,010 (2.55)	39 (2.77)	2584 (16.31)*	5356 (4.16)*	1377 (4.47)*	1563 (4.74)*
Characteristics of the 10% PBS random sample of Australians aged 60 years or over during 2022 dispensed antiviral treatment for COVID-19						
	*N* = 41,362	*N* = 120	*N* = 1728	*N* = 11,755	*N* = 2895	*N* = 3492
Age in years, mean ± SD	76.24 ± 8.27	75.06 ± 8.24	79.56 ± 9.10*	76.80 ± 8.72*	76.42 ± 8.85	77.83 ± 8.40*
Female	23,149 (55.97)	69 (57.50)	1038 (60.07)*	7749 (65.92)*	1930 (66.67)*	2298 (65.81)*
Rx-risk score, mean ± SD	4.09 ± 2.36	4.22 ± 2.26	4.60 ± 2.36*	4.81 ± 2.41*	4.91 ± 2.40*	4.89 ± 2.50*
Died	1237 (2.99)	9 (7.50)*	255 (14.76)*	572 (4.87)*	131 (4.53)*	141 (4.04)*

* *p* < 0.01 compared with the remainder of the population not dispensed the relevant psychotropic medication.

Of the 14,010 participants who died, 1237 (8.83%) had received antiviral treatment for COVID-19 from March to December 2022. Antiviral treatment for COVID-19 decreased the odds of death after statistical adjustment for age, sex and simplified Rx-Risk score (OR = 0.70, 99% CI = [0.65, 0.76]). Treatment for COVID-19 and antipsychotic medications interacted significantly (*p* < 0.01) in the model to predict death (*p* < 0.01). The adjusted interaction terms between antiviral dispensing for COVID-19 and other psychotropic medications were not significant (*p* > 0.01) (Table 1).

Among people dispensed antiviral treatment for COVID-19, there was an age, sex and simplified Rx-Risk score-adjusted excess mortality among participants dispensed lithium (OR = 3.50, 99% CI = [1.36, 8.98]), antipsychotics (OR = 4.98, 99% CI = [4.05, 6.13]), antidepressants (OR = 1.84, 99% CI = [1.57, 2.15]) and anxiolytics (OR = 1.40, 99% CI = [1.09, 1.80]), but not hypnotics (OR = 1.02, 99% CI = [0.80, 1.31]).

## Discussion

The occurrence of COVID-19 antiviral dispensing and death was higher among individuals dispensed antipsychotics, antidepressants, anxiolytics and hypnotics compared with people not dispensed these drugs. The odds of death were lower among participants dispensed COVID-19 antivirals, but higher for those dispensed antidepressants, anxiolytics, hypnotics and, most markedly, antipsychotics. The dispensing of antiviral treatment for COVID-19 had a significant impact on the odds of death among those dispensed antipsychotics.

Our findings are consistent with those of other studies showing that individuals receiving treatment for a mental health disorder have higher mortality (Nemani et al., 2021), including death after infection with COVID-19 (Vai et al., 2021). We confirmed that the dispensing of antiviral treatment for COVID-19 was associated with decreased mortality, and that this effect was not modified by the dispensing of lithium, antidepressants, anxiolytics or hypnotics. The finding that the use of COVID-19 antivirals decreased mortality markedly among those dispensed antipsychotics was unexpected and may indicate that people with psychotic illness who fill their scripts are more likely to adhere to other medical interventions that contribute to decrease mortality. Nonetheless, mortality among older adults dispensed psychotropics (particularly antipsychotics) was disproportionally high and suggests that the increasing availability of antiviral treatments may be insufficient to shift the excess mortality associated with COVID-19 among people with severe mental disorders.

### Limitations

We had access to a large community-representative sample of older Australians, but cannot be certain that our results would apply equally to other settings or to younger populations. However, as COVID-19-associated death is higher in later life, studies designed to investigate the effect of exposures on mortality are particularly relevant for older people. While there is evidence supporting agreement between the dispensing and consumption of medications, poor adherence could lead to misclassification and loss of power. Residual confounding and confounding by unmeasured factors cannot be dismissed (e.g. vaccination status), although existing evidence shows that COVID-19 antiviral treatment reduces hospitalisations and death of both vaccinated and non-vaccinated individuals (Hammond et al., 2022). We had no access to the specific causes of death.

## Conclusion

Our results highlight the need for a comprehensive approach to address the excess mortality associated with severe mental illness and COVID-19 among older adults. Mitigating the health hazards faced by this vulnerable population may require ongoing vigilance and the use of effective preventive measures that go beyond the increasing availability of antiviral treatments for COVID-19.
